# A Case of Brugada Syndrome… or Something Else? Behind Type 1 Brugada Pattern

**DOI:** 10.1002/ccr3.73042

**Published:** 2026-06-23

**Authors:** Antonio Scarà, Alessio Borrelli, Silvio Romano, Luigi Sciarra

**Affiliations:** ^1^ San Carlo di Nancy GVM‐Hospital Rome Italy; ^2^ Department of Life, Health and Environmental Sciences University of L'Aquila L'Aquila Italy

**Keywords:** Brugada‐like ECG pattern, cardiac MRI, fever‐induced ECG changes, scarring cardiomyopathy, syncope, ventricular arrhythmia

## Abstract

Although Brugada syndrome has traditionally been considered a primary electrical disease, accumulating evidence supports the presence of subtle structural abnormalities, particularly involving the right ventricular outflow tract. Nevertheless, the identification of overt myocardial scar and biventricular arrhythmogenic substrate should prompt consideration of an alternative diagnosis. We report the case of a 51‐year‐old man presenting with syncope during a febrile illness and a transient spontaneous type‐1 Brugada ECG pattern. Initial evaluation was unremarkable. However, Holter monitoring revealed multifocal ventricular ectopy, including non‐sustained ventricular tachycardia (NSVT) with both left and right bundle branch block morphologies, suggesting biventricular origin. Cardiac magnetic resonance showed a non‐ischemic subepicardial scar, and electroanatomical mapping identified additional right ventricular abnormalities. Programmed stimulation induced sustained ventricular arrhythmias. These findings supported the presence of an underlying biventricular structural scar cardiomyopathy presenting with a fever‐induced type‐1 Brugada ECG pattern. The patient underwent transvenous ICD implantation. This case highlights the importance of a comprehensive multimodal diagnostic approach.

## Key Clinical Message

A fever‐induced type 1 Brugada ECG pattern does not necessarily indicate isolated Brugada syndrome. The presence of ventricular arrhythmias or imaging abnormalities should prompt comprehensive evaluation for an underlying structural cardiomyopathy to ensure appropriate diagnosis and risk stratification.

## 1 Introduction

A type‐1 Brugada electrocardiographic (ECG) pattern may be unmasked by fever and strongly suggest Brugada syndrome (BrS). Similar ECG features may also arise in the context of metabolic disturbances, ischemia, or structural myocardial disease. Distinguishing true Brugada syndrome from alternative conditions presenting with a Brugada‐like ECG pattern is crucial, as risk stratification, prognosis, and therapeutic strategies differ substantially [1].

## 2 Case History/Examination

A previously healthy 51‐year‐old man experienced a sudden loss of consciousness during a febrile illness (38.8°C). The episode occurred without chest pain or palpitations. Family history was negative for sudden cardiac death.

Initial ECG showed a spontaneous type‐1 Brugada pattern in leads V1–V2 (Figure 1a). Laboratory tests revealed elevated inflammatory markers but no myocardial injury. Echocardiography showed normal biventricular function.

**FIGURE 1:**
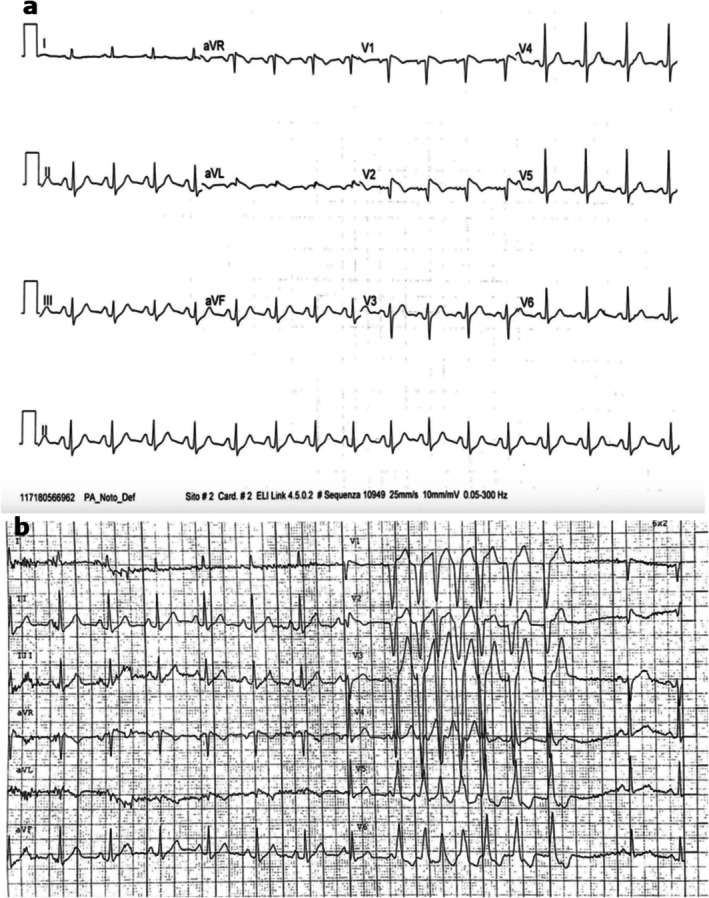
(a) The figure illustrates the electrocardiogram obtained in the emergency department. The patient was febrile at the time of recording. A coved‐type ST‐segment elevation in leads V1–V2 is evident, consistent with a type 1 Brugada pattern. (b) The figure illustrates an electrocardiogram obtained during the initial hospitalization. A non‐sustained ventricular tachycardia exhibiting a left bundle branch block morphology was recorded.

During hospitalization, telemetry recorded non‐sustained ventricular tachycardia (NSVT) with left bundle branch block morphology (Figure 1b), suggesting right ventricular origin.

## 3 Differential Diagnosis, Investigations, and Treatment

Initial diagnosis of Brugada syndrome was considered. Since the type‐1 Brugada pattern had been documented only during the febrile episode, a 12‐lead Holter monitor with modified precordial lead positioning was performed to assess whether spontaneous type‐1 ECG changes were present under afebrile conditions [2]. This was considered particularly relevant because the combination of fever, syncope, and a type‐1 Brugada ECG pattern may identify patients at higher arrhythmic risk and warrants careful evaluation for spontaneous ECG abnormalities [3]. Subsequent 24‐h Holter monitoring revealed frequent multifocal ventricular ectopy, including NSVT with both left and right bundle branch block morphologies (Figure 2), suggesting biventricular origin.

**FIGURE 2:**
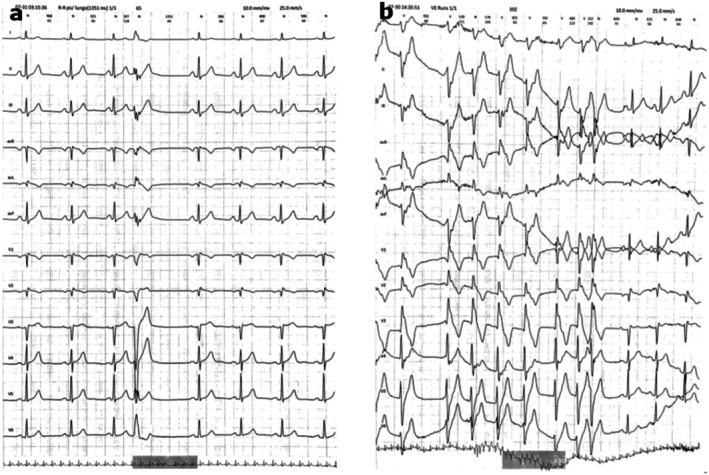
The figure illustrates two electrocardiographic tracings recorded by 12‐lead Holter monitoring. Panel (a) shows a premature ventricular beat with a left bundle branch block morphology. Of note, fragmented QRS complexes are visible in the precordial leads, suggesting delayed and heterogeneous ventricular conduction related to the underlying arrhythmogenic substrate. Panel (b) shows a non‐sustained ventricular tachycardia exhibiting a right bundle branch block morphology.

Cardiac magnetic resonance imaging demonstrated subepicardial late gadolinium enhancement in the left ventricle, consistent with myocardial scar (Figure 3).

**FIGURE 3:**
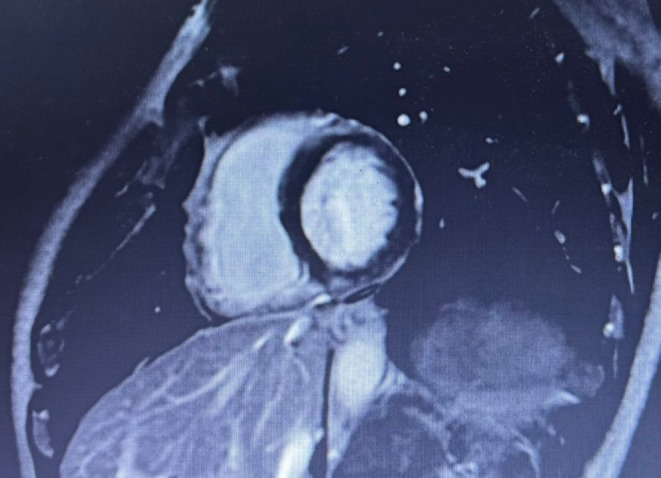
The figure shows a contrast‐enhanced cardiac MRI performed in the patient. A scar is evident in the basal posterolateral region, with a predominantly subepicardial distribution.

Electroanatomical mapping was conducted using contact force monitoring to improve voltage map accuracy and minimize false‐positive scar detection [4]. This revealed low‐voltage areas in the right ventricular outflow tract (Figure 4, upper panel).

**FIGURE 4:**
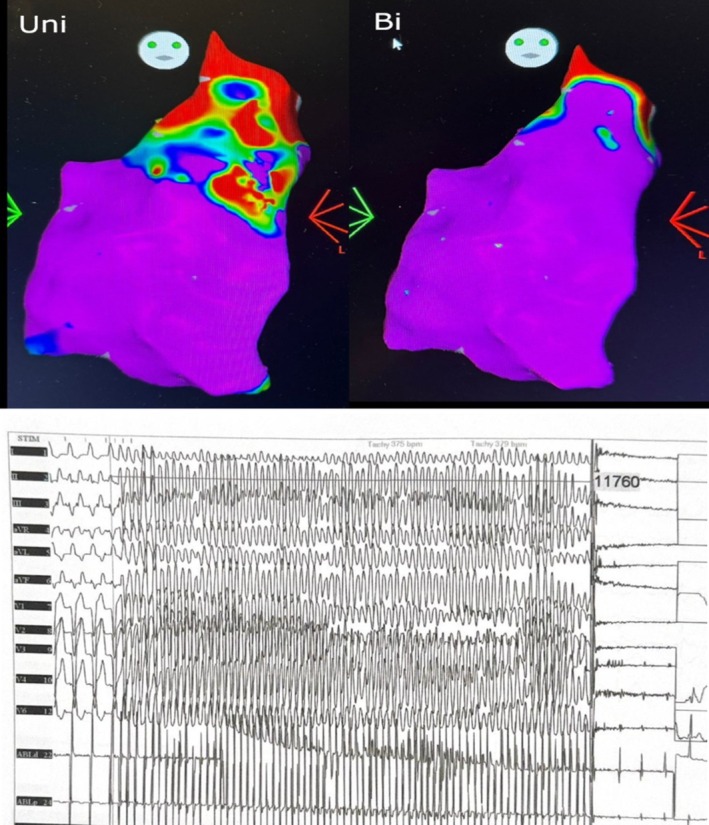
The figure shows, in the upper panel, the patient's right ventricular CARTO electroanatomical map. An electrical abnormality is clearly evident in the epicardial region of the anterior outflow tract, as indicated by the unipolar (UNI) voltage color scale. The lower panel demonstrates the inducibility of a rapid ventricular tachycardia/ventricular flutter through programmed ventricular stimulation with a double extrastimulus.

Programmed ventricular stimulation induced sustained ventricular arrhythmias (Figure 4, lower panel).

## 4 Conclusion and Results (Outcome and Follow‐Up)

A transvenous implantable cardioverter‐defibrillator (ICD) was implanted after failed subcutaneous screening. At follow‐up, the patient remained clinically stable with no recurrence of syncope or sustained ventricular arrhythmias.

## 5 Discussion

Fever is a well‐recognized trigger of type‐1 Brugada electrocardiographic patterns and may unmask latent abnormalities in cardiac sodium channel function. Previous studies have demonstrated that fever‐induced Brugada patterns are associated with an increased risk of ventricular arrhythmias and may represent the first clinical manifestation of Brugada syndrome [5, 6, 7, 8]. For this reason, the identification of a type‐1 Brugada pattern during a febrile illness warrants careful clinical evaluation and risk stratification.

However, the distinction between Brugada syndrome and structural cardiomyopathies presenting with a Brugada‐like electrocardiographic phenotype may be challenging. Although Brugada syndrome was initially regarded as a pure channelopathy, accumulating evidence has demonstrated the presence of structural and electrophysiological abnormalities, particularly involving the right ventricular outflow tract. Therefore, the mere presence of a type‐1 Brugada ECG pattern does not necessarily establish the diagnosis of isolated Brugada syndrome, especially when additional evidence of myocardial disease is present.

In the present case, several findings suggested an underlying structural arrhythmogenic cardiomyopathy rather than isolated Brugada syndrome. Cardiac magnetic resonance revealed a non‐ischemic subepicardial scar involving the basal posterolateral left ventricular wall. Furthermore, 12‐lead Holter monitoring documented ventricular arrhythmias with morphologies suggesting origins from both ventricles, while electroanatomical mapping demonstrated extensive right ventricular abnormalities involving the outflow tract region. Of note, fragmented QRS complexes were also observed on surface ECG recordings, a finding that may reflect delayed and heterogeneous ventricular conduction associated with myocardial scar and arrhythmogenic substrate [9].

Taken together, these findings support the presence of a broader biventricular structural substrate capable of explaining both the electrocardiographic abnormalities and the ventricular arrhythmias observed in our patient. Importantly, the extent of structural abnormalities identified in both ventricles exceeded that typically described in Brugada syndrome and therefore favored an alternative cardiomyopathic substrate. The fever‐induced type‐1 Brugada pattern was therefore interpreted as an epiphenomenon occurring in the setting of an underlying structural scar cardiomyopathy rather than as evidence of isolated Brugada syndrome.

This case highlights the importance of comprehensive phenotypic characterization in patients presenting with fever‐induced type‐1 Brugada patterns, particularly when ventricular arrhythmias or imaging abnormalities are present. In such cases, multimodality evaluation including cardiac magnetic resonance imaging and electroanatomical mapping may be crucial for identifying an underlying structural cardiomyopathy and guiding appropriate risk stratification and management.

## Author Contributions

**Alessio Borrelli:** visualization, validation. **Antonio Scarà:** conceptualization, project administration, writing – original draft, writing – review and editing. **Silvio Romano:** supervision, writing – review and editing. **Luigi Sciarra:** writing – review and editing, supervision.

## Funding

The authors have nothing to report.

## Ethics Statement

The authors have nothing to report.

## Consent

Written informed consent was obtained from the patient for publication of this case report and accompanying images, in accordance with *Clinical Case Reports* journal requirements.

## Conflicts of Interest

The authors declare no conflicts of interest.

## Data Availability

Data are available from the corresponding author upon reasonable request.
